# To Stamp out Tuberculosis

**Published:** 1902-07

**Authors:** 


### Abstracts and Selections.

### To Stamp Out Tuberculosis.

A paper in the March number of the *St. Paul Medical Journal* contains much valuable advice with reference to the preventive treatment of tuberculosis. The battle against the disease, the writer says, must be fought by the public itself and not by physicians. He thinks that within a short time consumption would be as rare in this country as is the black death if proper preventive measures were adopted. He suggests that the public should demand that a national board of health be established, that information relating to the infectiousness of tuberculosis should be disseminated, that the laws against spitting in public places should be enforced, that all meat used for food should be rigidly inspected, that sanatoria should be provided for the consumptive poor, and that the formation of societies for the enforcement of sanitary regulations should be encouraged. R.

